# Excipients

**Published:** 1878-01

**Authors:** 


### EXCERPTA.

Excipients.—Before the days of glycerine, pill makers, as well as pill takers, had good grounds to complain. The former, because his stock of ready-made pills had to be very frequently renewed, in order to present a soluble article; the latter, because it was not unfrequently the case to find, instead of pills swallowed, it was something nearer the insolubility of bullets, for, despite all the solvent fluids of the stomach, they persisted in passing off whole, undissolved, unchanged. The universal excipient was gum arabic, tragacanth and syrup, water, or a combination of them—confection rose. To this subject, of late years, more interest has been paid than before, which has given rise to a number of suggestions as to excipients.

The writer, having a considerable experience at the compounding counter for a number of years, has tried almost every excipient published, and tested many of his own. He came to the conclusion that, at the prescription counter, three or four excipients are absolutely necessary for elegant work:

1. A liquid containing glycerine 3 parts, alcohol 1 part. It is best to keep this in a wide-mouthed bottle. The cork should be perforated by two holes; one for an exit tube, another for a bulb, so that by pressure upon the bulb the liquid will come out of the tube, drop by drop.

2. Soft excipient, containing grs. lxx tragacanth and ℥ j glycerine.

3. Hard excipient. Tragacanth ℥ ij and glycerine ℥ j.

4. Adhesive excipient, composed of balsam fir 1 part, rosin 1¼ parts, wax 1¼ parts.

It would be too tedious for me to describe the merits of each of these separately; but one in regular practice, who furnishes himself with this list of excipients always on hand for immediate use, will in time find they are indispensable. As to ready-made pills, glycerine, unquestionably, is the only article which will keep them soft and soluble for any length of time.

It is true there are many other articles which the expert brings to hand in order to bring his mass to the nice condition called pillular. For example: iodide of iron pills need a little thickening with pulv. glycyr., before adding the adhesive excipient; some gum resins need a little alkali, soap or carb. potass.; oleo resins need a little magnesia, copabia (about one-sixteenth of its weight calcined or its own weight of carbonate). Articles of fatty nature, also croton oil, creosote, etc., crumb of bread.

The great point in nice pill making is to have a well educated idea of firmness and adhesiveness. Plasticity is a well-balanced union of these two, I might say opposite, forces. Its presence gives the perfection to pill mass, sufficient softness to roll out with ease, with firmness requisite to enable the pill to maintain its correct shape.—*Druggist and Chemist.*

